# Supplementary material for: Cyanobacterial Harmful Bloom Lipopolysaccharides Induce Pro-Inflammatory Effects in Immune and Intestinal Epithelial Cells In Vitro
Source: Toxins (Basel). 2023 Feb 21;15(3):169. doi: 10.3390/toxins15030169 (PMC10058507; doi:10.3390/toxins15030169)
Supplement: Supplementary file 1 [file toxins-15-00169-s001.zip › toxins-2164833-supplementary.pdf]

toxins

MDPI

Supplementary Materials: Cyanobacterial Harmful Bloom Lipopolysaccharides Induce Pro-Inflammatory Effects in Immune and Intestinal Epithelial Cells *In Vitro*

Veronika Skočková, Ondřej Vašíček, Eliška Sychrová, Iva Sovadinová, Pavel Babica and Lenka Šindlerová

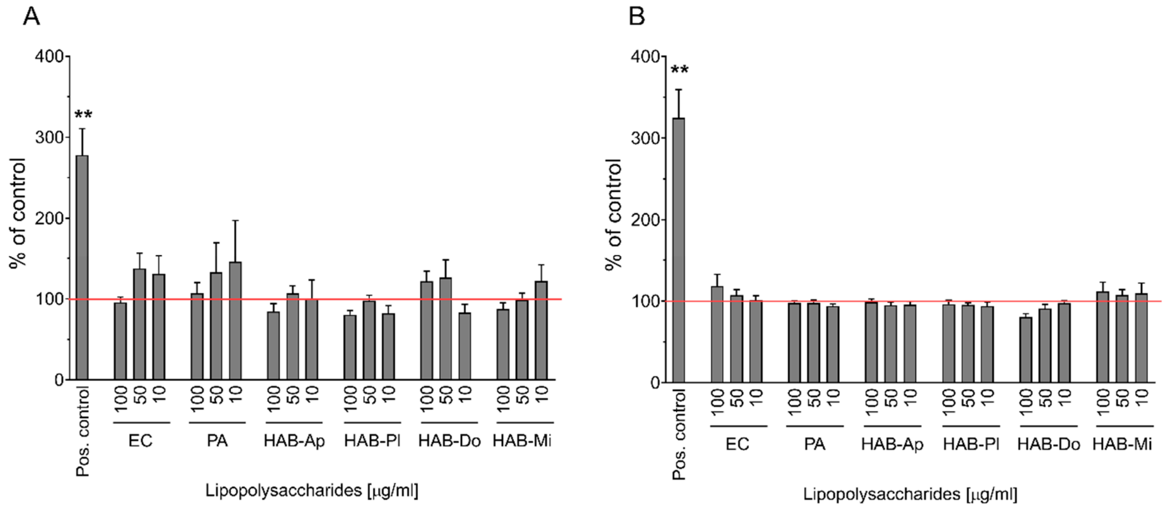

Figure S1. Release of LDH into the medium – the cytotoxicity assay. LDH activity in medium of differentiated Caco-2 (A) and HT-29 (B) cells exposed to LPS CyanoHABs (10, 50, and 100 µg/ml) for 24 h. Untreated cells were used as a negative control. *Escherichia coli* and *Pseudomonas aeruginosa* LPS were used as a positive control of the LPS effect, untreated cells lysed by supplier-provided lysis buffer were used as a positive control of the method. Data were converted to percent of the negative control (100% level denoted by the red line) and expressed as the mean ±SEM. Data were statistically analysed using One sample t-test to hypothetical value 100. n≥3, * $p < 0.05$, ** $p < 0.01$.

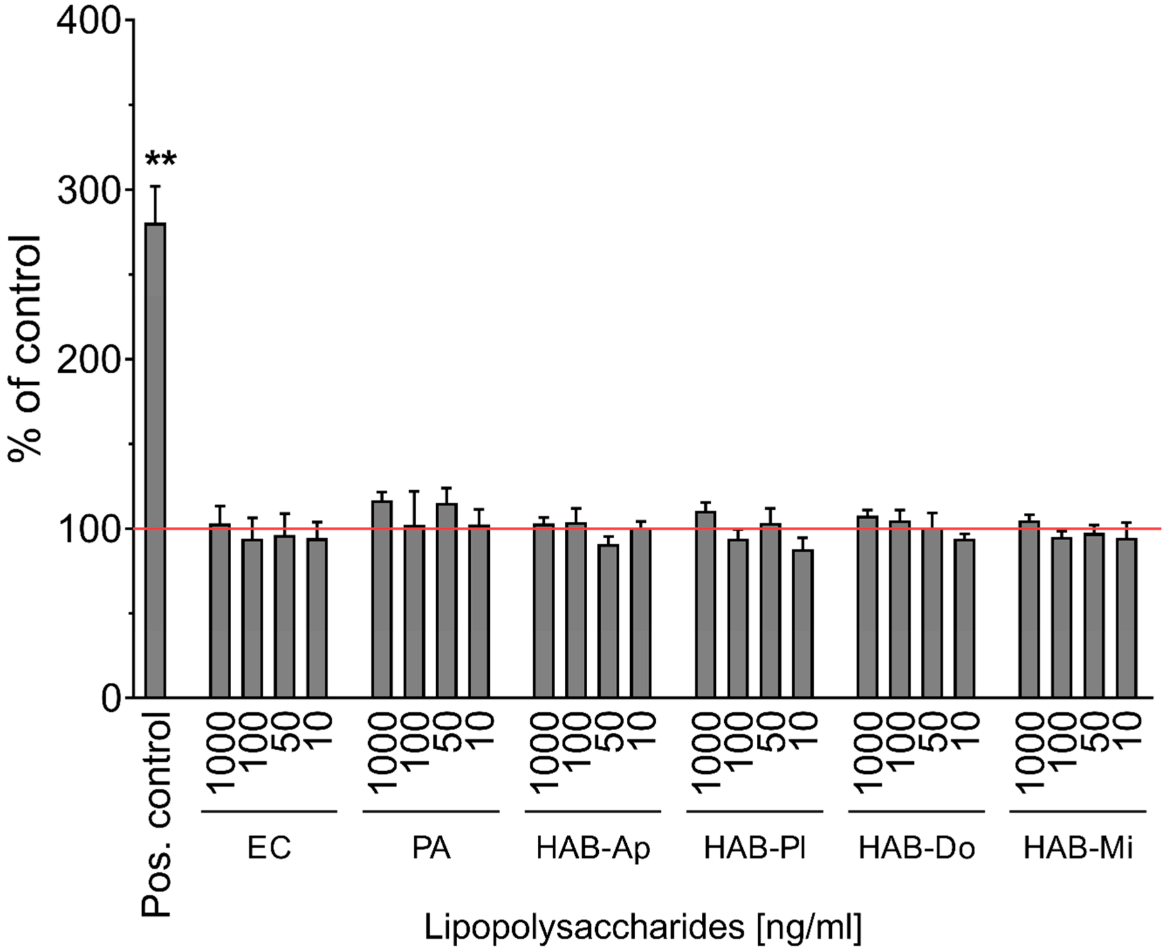

Figure S2. Release of LDH into the medium – the cytotoxicity assay. LDH activity in medium of RAW 264.7 cells exposed to LPS CyanoHABs (10, 50, 100, and 1 000 ng/ml) for 24 h. Untreated cells were used as a negative control. *Escherichia coli* and *Pseudomonas aeruginosa* LPS were used as a positive control of the LPS effect, untreated cells lysed by supplier-provided lysis buffer were used as a positive control of the method. Data were converted to percent of the negative control (100% level denoted by the red line) and expressed as the mean ±SEM. Data were statistically analysed using One sample t-test to hypothetical value 100. $n \geq 3$, * $p < 0.05$, ** $p < 0.01$.

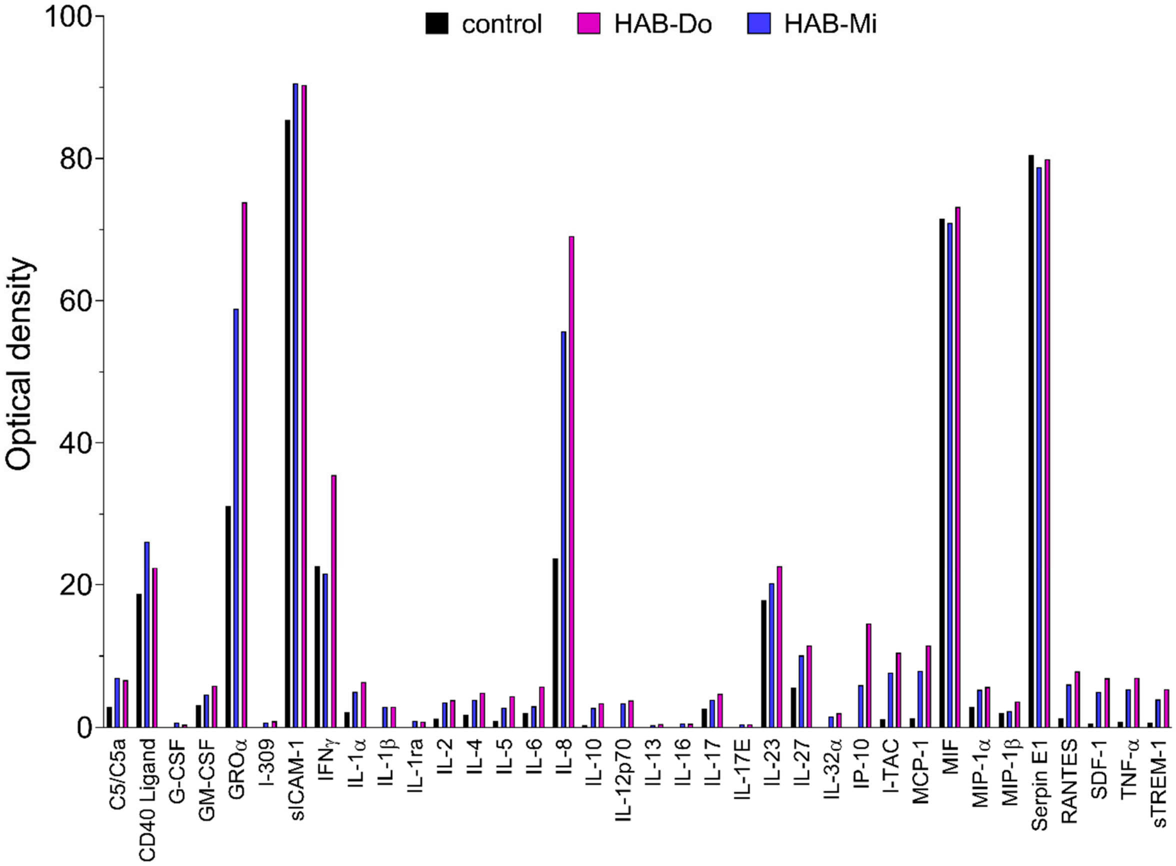

Figure S3. Cytokine array. Medium from 4-7 independent experiments with differentiated Caco-2 cells exposed to 100 μg/ml HAB-Mi and HAB-Do for 24 hours was collected, pooled, and the cytokine array was performed. Optical density of each dot on the membrane was determined, duplicates were averaged and the obtained values were plotted.

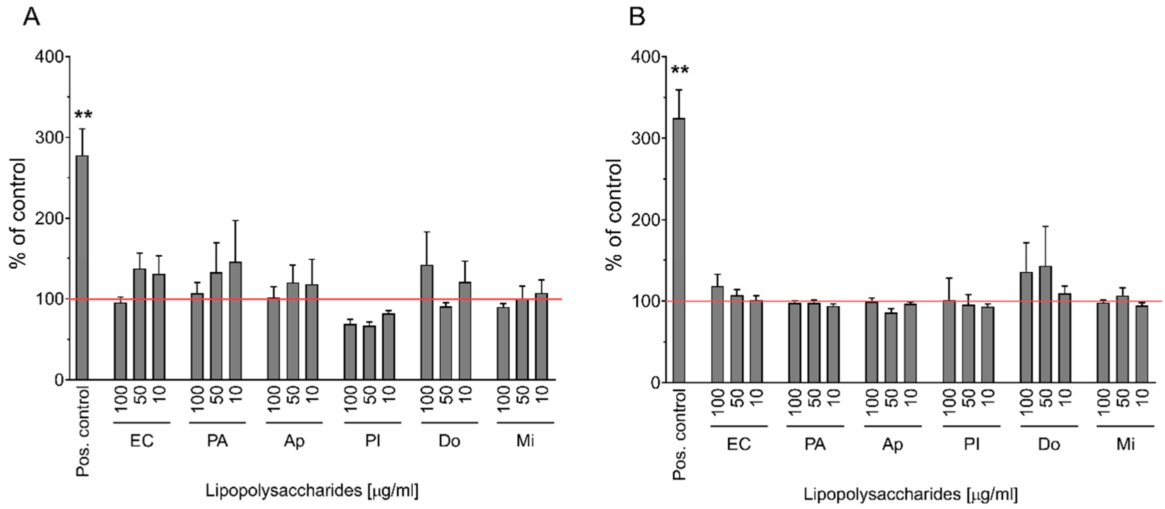

Figure S4. Release of LDH into the medium – the cytotoxicity assay. LDH activity in medium of differentiated Caco-2 (A) and HT-29 (B) cells exposed to LPS isolated from cyanobacterial cultures (10, 50, and 100 μg/ml) for 24 h. Untreated cells were used as a negative control. *Escherichia coli* and *Pseudomonas aeruginosa* LPS were used as a positive control of the LPS effect, untreated cells lysed by supplier-provided lysis buffer were used as a positive control of the method. Data were converted to percent of the negative control (100% level denoted by the red line) and expressed as the mean ±SEM. Data were statistically analysed using One sample t-test to hypothetical value 100. n≥3, * $p < 0.05$, ** $p < 0.01$.

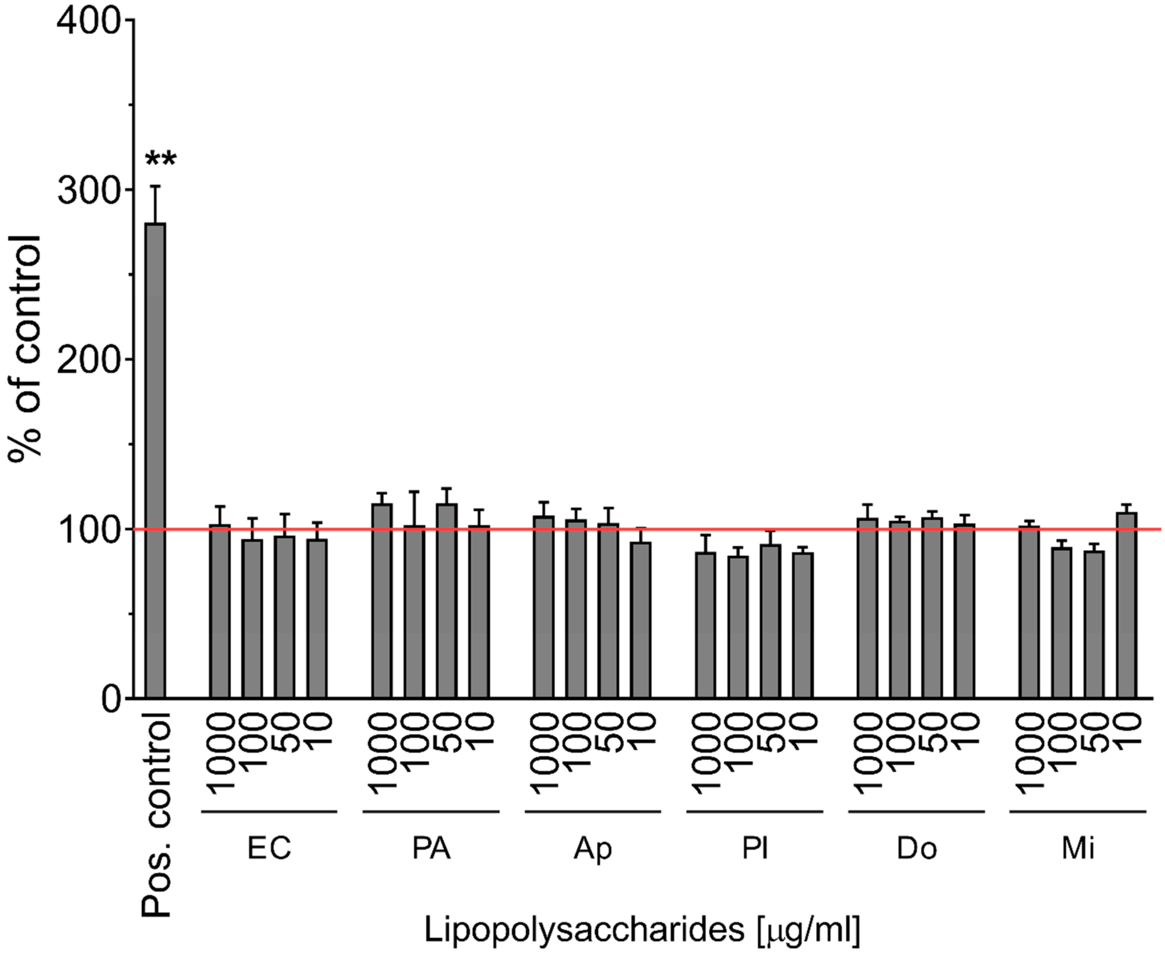

Figure S5. Release of LDH into the medium – the cytotoxicity assay. LDH activity in medium of RAW 264.7 cells exposed to LPS isolated from cyanobacterial cultures (10, 50, 100, and 1 000 ng/ml) for 24 h. Untreated cells were used as a negative control. *Escherichia coli* and *Pseudomonas aeruginosa* LPS were used as a positive control of the LPS effect, untreated cells lysed by supplier-provided lysis buffer were used as a positive control of the method. Data were converted to percent of the negative control (100% level denoted by the red line) and expressed as the mean ±SEM. Data were statistically analysed using One sample t-test to hypothetical value 100. n≥3, * $p < 0.05$, ** $p < 0.01$.

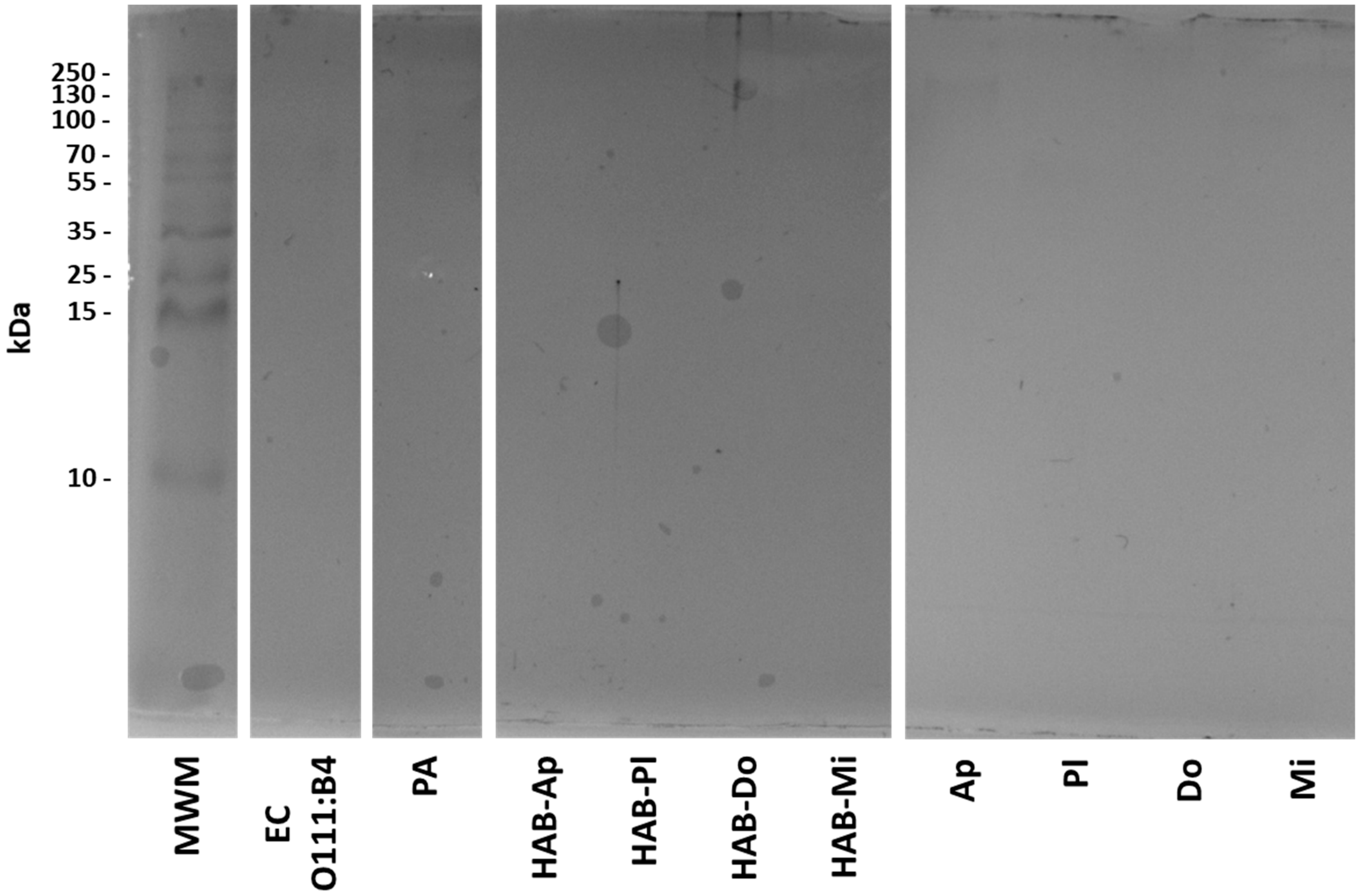

Figure S6. SDS-PAGE of LPS followed by Coomassie Brilliant Blue staining. LPS were isolated from cyanobacterial water blooms (CyanoHAB) dominated by *Aphanizomenon* (HAB-Ap), *Planktothrix* (HAB-Pl), *Dolichospermum* (HAB-Do) and *Microcystis* (HAB-Mi), or from cultured cyanobacteria: Ap – *Aphanizomenon* PCC7905, Pl – *Planktothrix* NIVA/CYA 126/8, Do – *Dolichospermum* CCALA007, Mi – *Microcystis* PCC7806. Positive controls: EC – LPS from *Escherichia coli* serotype O111:B4 and PA –LPS from *Pseudomonas aeruginosa.* MWM – protein molecular weight marker.
